# Obesity-Related Genetic Variants and Hyperuricemia Risk in Chinese Men

**DOI:** 10.3389/fendo.2019.00230

**Published:** 2019-04-12

**Authors:** Zhimin Ma, Yunfeng Wang, Chaonan Xu, Feiling Ai, Ling Huang, Jieping Wang, Ji Peng, Yanming Zhou, Meihua Yin, Shan Zhang, Xinghua Yang

**Affiliations:** ^1^School of Public Health, Capital Medical University, Beijing, China; ^2^Shenzhen Center for Chronic Disease Control, Shenzhen, China; ^3^Beijing Municipal Key Laboratory of Clinical Epidemiology, Beijing, China; ^4^Fu Xing Hospital, Capital Medical University, Beijing, China

**Keywords:** obesity, metabolic syndrome, single nucleotide polymorphisms, hyperuricemia, gene

## Abstract

**Objective:** Obesity/metabolic syndrome and hyperuricemia are clinically associated; however, the association of obesity/metabolic syndrome-related genetic variants with hyperuricemia is not clear. Therefore, we assessed this association in Chinese men diagnosed with hyperuricemia in comparison to a non-hyperuricemia group.

**Methods:** We genotyped 47 single nucleotide polymorphisms (SNPs) previously identified to be associated with obesity or metabolic syndrome in 474 adult males (aged ≥ 18 years) using multiplex polymerase chain reaction. Multivariate logistic regression was used to investigate the association between the genetic variations and hyperuricemia. Stratified analyses were applied to further assess the associations.

**Results:** The obesity-related SNP in MSRA rs545854 significantly affected serum uric acid levels. In addition, the G-allele of rs545854 was positively associated with the risk of hyperuricemia [odds ratio (OR) = 2.80, 95% confidence interval (CI) = 1.19–6.64, *P* = 0.0188]. After adjusting the model for body mass index and central obesity, rs545854 was shown to be an independent factor increasing the risk of hyperuricemia (OR = 2.81, 95%CI = 1.18–6.70, *P* = 0.0196). Stratified analyses also showed a significant association between rs545854 and hyperuricemia among meat eaters (OR = 2.62, 95%CI = 1.09–6.26, *P* = 0.0308).

**Conclusion:** The obesity-related SNP rs545854 was correlated with the serum uric acid level and risk of hyperuricemia in a male Chinese population. Therefore, men carrying this SNP could benefit from limiting their meat consumption to prevent hyperuricemia. These findings suggest an underlying genetic link between obesity and hyperuricemia worthy of further exploration.

## Introduction

Serum uric acid (SUA) is a final product of the metabolic breakdown of purine oxidation (1). Since humans lack the gene for uricase that converts uric acid into a soluble form, the human uric acid level tends to be higher than that of other mammals that produce uricase (2). Indeed, an elevated SUA level was identified as the cause of gout in the early 1800s (3), and has since been associated with a wide range of health outcomes, including renal disorders, cardiovascular diseases, metabolic syndrome (MS), diabetes, and cancer (4–6). In addition, the high prevalence of hyperuricemia represents a great public health concern. The prevalence of hyperuricemia was reported at 21.0% in the total population, including 21.2% for men and 21.6% for women, in the USA (7). Moreover, the prevalence of hyperuricemia in Chinese men and women was recently reported at 19.4 and 7.9%, respectively (8).

The varying levels of uric acid in human populations can be attributed to environmental factors such as diet, including dietary deficiency of uricase, and genetic factors (9). In addition, hyperuricemia is commonly observed in patients with obesity and MS (10). Although the increase in serum SUA has generally been considered to be secondary to these conditions (11, 12), recent studies suggest that it may play a more direct and contributory role (13). These conflicting findings indicate that potential associations between hyperuricemia and obesity or MS are related to a shared genetic background.

With the advent of new molecular analysis tools, genetic information is now widely used to assess causality in the pathogenesis of complex disorders (14). Genetic variants are inherited independent of potential confounding factors, since alleles are allocated randomly during gamete formation (15). In addition, numerous single nucleotide polymorphisms (SNPs) have been reported to be associated with overweight/obesity, hypertension, and other MS components in Asian populations as in Caucasian and Hispanic-American Populations (16–18). However, few studies have explored whether these SNPs are associated with hyperuricemia in Chinese populations. Accordingly, the aim of this study was to investigate the genetic link between MS components and hyperuricemia in Chinese men. Moreover, we further investigated the influence of key clinical and demographic characteristics on mediating the association between these SNPs and hyperuricemia. These findings should shed light on the underlying biological mechanisms mediating obesity/MS and hyperuricemia.

## Methods

### Study Population

A cross-sectional study was conducted at the Physical Examination Center of Fu Xing Hospital between April and May 2013. The records of a total of 474 male adults (aged 18 years or older) who underwent an annual health examination at this center were reviewed for inclusion in the study. Among these patients, 66 individuals were diagnosed with hyperuricemia based on the definition of an SUA level > 7.0 mg/dL in men (4). As non-hyperuricemia group, a total of 408 men without hyperuricemia were included based on the following criteria: no history of hyperuricemia, gout, MS, kidney disease, or cardiovascular disease. As for the study participants, we excluded female participants since metabolic or additional hormonal effects can influence the SUA levels as well as the risk of hyperuricemia in women (19). In addition, individuals with type 2 diabetes or those that had taken any agents that lower SUA levels in the last 6 months were excluded from the study.

The study protocol was approved by the ethics committee of Fu Xing Hospital (Beijing, China), and written informed consent was provided by each participant.

### Anthropometric and Demographic Characteristics

Detailed information regarding demographic characteristics, dietary habits, lifestyle factors, and health conditions was obtained through a structured questionnaire conducted during face-to-face interviews. For example, salt intake was categorized into three classes: low-salt diet, normal-salt diet, and high salt diet. In addition, exercise was categorized into two classes: < 1 h/week and >1 h/week.

Anthropometric and biochemical characteristics of the subjects were collected by well-trained staff. Height, weight, and waist circumference (WC) were measured for each participant while dressed in their regular indoor clothes without shoes. Body mass index (BMI) was calculated as the weight in kilograms divided by height in meters squared, and was used to classify subjects into the following categories according to the accepted Chinese BMI standard: underweight (BMI < 18.5 kg/m^2^), normal weight (18.5 ≤ BMI < 24.0 kg/m^2^), overweight (24.0 ≤ BMI < 28.0 kg/m^2^), and obese (BMI ≥ 28.0 kg/m^2^) (20, 21). Central obesity was defined as a WC ≥ 90 cm in Chinese men (22).

### Laboratory Examination and Clinical Assessment

SUA, creatinine, blood glucose, triglycerides, total cholesterol (TC), low-density lipoprotein cholesterol (LDL-C), and high-density lipoprotein cholesterol (HDL-C) levels were measured at the hospital's central certified biochemical laboratory. High blood glucose was identified as a fasting blood glucose level ≥ 7.0 mmol/L. Hypertension was defined according to self-reported history and/or systolic pressure ≥ 140 mmHg or diastolic pressure ≥ 90 mmHg. Low HDL-C was identified as a fasting HDL-C level ≤ 0.9 mmol/L, and high LDL-C was defined as a fasting LDL-C level ≥ 4.1 mmol/L. Hypertriglyceridemia was regarded as fasting triglycerides ≥ 2.3 mmol/L. Hypercholesterolemia was defined as a fasting TC level ≥ 6.2 mmol/L. Non-alcoholic fatty liver disease (NAFLD) was diagnosed on the basis of established guidelines from the American Association for the Study of Liver Diseases (23).

### Genotyping

We searched the literature and SNP databases to identify candidate SNPs associated with obesity or MS for assessment. A total of 47 SNPs reportedly associated with overweight/obesity, hypertension, and MS in Asian populations were genotyped (16, 17, 24). Genomic DNA was extracted from peripheral leukocytes using phenol chloroform. The primers and probes for SNP amplification were designed using Sequenom Assay Design 3.1 software (Sequenom, Inc., San Diego, CA, USA). SNP genotyping was conducted using the Mass Array system (Sequenom, Inc.) based on high-throughput multiplex polymerase chain reaction (PCR) amplification of target fragments in a 384-well PCR plate. The PCR products were subjected to uric acidification and primer single-base extension reaction. Alleles were then detected by matrix-assisted laser desorption time-of-flight mass spectrometry (Sequenom, Inc.), and mass spectra analysis was conducted with Mass Array Typer 4.0 software (Sequenom, Inc.).

### Statistical Analysis

All statistical analyses were conducted using SAS software version 9.4 (SAS Institute Inc). Genotype data were examined for Hardy-Weinberg equilibrium using a chi-square test. Continuous and categorical variables are presented as mean ± standard deviation and numbers with percent frequency, respectively. Differences between the hyperuricemia and non-hyperuricemia groups were assessed by Student's *t*-test for normally distributed variables and with the chi-square test (or Fisher's exact test) for categorical variables. Differences in SUA levels among genotypes were evaluated by one-way analysis of variance. The inclusion criteria of candidate SNPs included conformance to Hardy-Weinberg equilibrium (*P* > 0.1), significant difference between hyperuricemia case and non-hyperuricemia groups (*P* < 0.1), and a difference in SUA levels across the genotype distribution (*P* < 0.1). Associations of risk alleles with hyperuricemia were assessed using multivariate logistic regression analysis. Independent associations were further evaluated after adjusting the model for BMI category and central obesity. Two-sided *P* < 0.05 were considered statistically significant. In addition, a series of stratified analyses were conducted to separately evaluate the correlation of SNP with SUA in Chinese men according to various demographic and anthropometric characteristics. Finally, the power of this study was estimated using Quanto software 1.2.4 (University of Southern California, USA).

## Results

### Baseline Characteristics

The demographic and biochemical characteristics of the male participants are summarized in Table 1. There was no difference in the average age between the hyperuricemia and non-hyperuricemia groups. However, significantly more of the men with hyperuricemia also showed hypertriglyceridemia and reported eating meat. Compared with the non-hyperuricemia group, men with hyperuricemia had a higher frequency of a BMI outside the healthy range, central obesity, hypertension, and NAFLD.

**Table 1:** Demographic and biochemical characteristics of study participants.

**Variables**	**Case**	**Control**	***P*-value**
	**(*n =* 66)**	**(*n =* 408)**	
Age, years	32.85 ± 8.07	31.36 ± 8.46	0.1691
**EDUCATION**			
High school and below, *n* (%)	2 (3.03%)	40 (9.80%)	0.1326
College or university, *n* (%)	48 (72.73%)	256 (62.75%)	
Graduate and above, *n* (%)	16 (24.24%)	112 (27.45%)	
**INCOME, RMB**			
< 3,000, *n* (%)	8 (12.31%)	64 (15.76%)	0.7616
3,000–5,000, *n* (%)	19 (29.23%)	98 (24.14%)	
5,000–10,000, *n* (%)	26 (40.00%)	160 (39.41%)	
>10,000, *n* (%)	12 (18.46%)	84 (20.69%)	
Work time, hours	3.39 ± 0.73	3.45 ± 0.67	0.3887
**EATING MEAT**			
Yes, *n* (%)	60 (92.31%)	319 (78.57%)	**0.0095**
No, *n* (%)	5 (7.69%)	87 (21.43%)	
**SALT INTAKE**			
Low-salt diet, *n* (%)	8 (12.12%)	88 (21.57%)	0.1582
Normal-salt diet, *n* (%)	36 (54.55%)	214 (52.45%)	
High-salt diet, *n* (%)	22 (33.33%)	106 (25.98%)	
**DRINKING**			
No, *n* (%)	52 (78.79%)	334 (81.86%)	0.5511
1–2 servings/week, *n* (%)	14 (21.21%)	74 (18.14%)	
**SMOKING**			
No, *n* (%)	33 (50.00%)	220 (53.92%)	0.2741
Passive smoke, *n* (%)	5 (7.58%)	45 (11.03%)	
Used to smoke, *n* (%)	4 (6.06%)	17 (4.17%)	
Occasionally smoking, *n* (%)	12 (18.18%)	40 (9.80%)	
Daily smoking, *n* (%)	12 (18.18%)	86 (21.08%)	
**EXERCISE**			
< 1 hour/week, *n* (%)	25 (38.46%)	112 (27.59%)	0.0731
>1 hours/week, *n* (%)	40 (61.54%)	294 (72.41%)	
**BMI CATEGORY**			
Underweight, *n* (%)	1 (1.52%)	9 (2.21%)	**<0.0001**
Normal-weight, *n* (%)	10 (15.15%)	205 (50.25%)	
Overweight, *n* (%)	25 (37.88%)	108 (26.47%)	
Obesity, *n* (%)	30 (45.45%)	86 (21.08%)	
**CENTRAL OBESITY**			
Yes, *n* (%)	49 (74.24%)	203 (49.75%)	**0.0002**
No, *n* (%)	17 (25.76%)	205 (50.25%)	
Creatinine (μmol/L)	67.22 ± 12.67	69.70 ± 11.94	0.1384
**HIGH BLOOD GLUCOSE**			
Yes, *n* (%)	58 (89.23%)	380 (93.60%)	0.1951^*^
No, *n* (%)	7 (10.77%)	26 (6.40%)	
**HYPERTENSION**			
Yes, *n* (%)	23 (34.85%)	80 (19.61%)	**0.0053**
No, *n* (%)	43 (65.15%)	328 (80.39%)	
**DECREASED HDL-C**			
Yes, *n* (%)	46 (69.70%)	287 (70.34%)	0.9152
No, *n* (%)	20 (30.30%)	121 (29.66%)	
**INCREASED LDL-C**			
Yes, *n* (%)	3 (4.55%)	5 (1.23%)	0.0862
No, *n* (%)	63 (95.45%)	403 (98.77%)	
**HYPERTRIGLYCERIDEMIA**			
Yes, *n* (%)	43 (65.15%)	357 (87.50%)	**<0.0001**
No, *n* (%)	23 (34.85%)	51 (12.50%)	
**HYPERCHOLESTEROLEMIA**			
Yes, *n* (%)	4 (6.06%)	21 (5.15%)	0.7580
No, *n* (%)	62 (93.94%)	387 (94.85%)	
**NAFLD**			
Yes, *n* (%)	50 (76.92%)	182 (44.83%)	**<0.001**
No, *n* (%)	15 (23.08%)	224 (55.17%)	

*Data are expressed as mean ± standard or n (%). Bold values indicate statistical significance*.

* Fisher exact test

*BMI, body mass index; HDL-C, high-density lipoprotein cholesterol; LDL-C, low-density lipoprotein cholesterol; NAFLD, non-alcoholic fatty liver disease*.

### Genotypic Frequencies of SNPs in Hyperuricemia and Non-hyperuricemia Groups

The 47 candidate SNPs were genotyped in all subjects. The frequencies of six SNPs showed notable heterogeneity (*P* < 0.1) between the hyperuricemia and non-hyperuricemia groups (Table 2): rs1294421, rs12970134, rs545854, rs713586, rs7359397, and rs987237, which genotyped within or near the genes lymphocyte antigen 86 (*LY86*), melanocortin 4 receptor (*MC4R*), methionine sulfoxide reductase (*MSRA*), adenylate cyclase 3 (*ADCY3*), SH2B adaptor protein 1 (*SH2B1*), and transcription factor AP-2 beta (*TFAP2B*), respectively. Of these six SNPs, the genetic variance of rs545854 and rs713586 was associated with SUA levels (*P* < 0.1). Since only rs545854 did not deviate significantly from Hardy-Weinberg equilibrium (*P* > 0.1), we further focused on the *MSRA* SNP rs545854 as a candidate for mediating the association between hyperuricemia and obesity/MS. The distributions of the remaining 41 SNPs in the hyperuricemia and non-hyperuricemia groups are presented in Table S1.

**Table 2:** Association of six SNPs with SUA.

**SNP**	**Locus**	**Genotype**	**Hyperuricemia**	**Non-hyperuricemia**	***X^**2**^***	***P*-value**	**SUA level**	***F***	***P*-value**	***P_***het***_***
rs1294421	*LY86*	TT	42 (63.64%)	253 (62.47%)	8.070	0.0177	334.93 ± 73.53	1.42	0.2418	0.13
		GT	15 (22.73%)	131 (32.35%)			327.34 ± 72.7			
		GG	9 (13.60%)	21 (5.18%)			351.2 ± 82.69			
rs12970134	*MC4R*	GG	44 (66.67%)	278 (68.14%)	4.797	0.0908	333.13 ± 73.55	0.79	0.4543	**< 0.05**
		GA	18 (27.27%)	123 (30.15%)			331.61 ± 72.06			
		AA	7 (6.06%)	4 (1.71%)			360.73 ± 111.75			
rs545854	*MSRA*	GG	23 (34.85%)	133 (33.25%)	5.207	0.0740	340.72 ± 67.91	5.43	0.0047	0.31
		CG	36 (54.55%)	177 (44.25%)			339.58 ± 78.62			
		CC	7 (10.60%)	90 (22.50%)			312.53 ± 69.41			
rs713586	*ADCY3*	TT	27 (41.54%)	117 (29.55%)	6.200	0.0451	344.07 ± 78.89	3.22	0.0408	**<0.05**
		CT	27 (41.54%)	230 (58.08%)			326.61 ± 66.91			
		CC	11 (16.92%)	49 (12.37%)			344.37 ± 87.69			
rs7359397	*SH2B1*	CC	53 (80.30%)	280 (68.63%)	4.72	0.0944	337.62 ± 73.092	2.13	0.1202	0.99
		CT	13 (19.70%)	115 (28.19%)			324.51 ± 77.28			
		TT	0 (0.00%)	13 (3.18%)			309.85 ± 58.93			
rs987237	*TFAP2B*	AA	37 (56.06%)	281 (69.38%)	5.7754	0.0557	329.28 ± 72.85	2.19	0.9656	0.97
		AG	25 (37.88%)	114 (28.15%)			340.04 ± 74.76			
		GG	4 (6.06%)	10 (2.47%)			353.07 ± 87.44			

*Data are expressed as mean ± standard or n (%). Bold values indicate statistical significance. SUA, serum uric acid; LY86, lymphocyte antigen 86; MC4R, melanocortin 4 receptor; MSRA, methionine sulfoxide reductase; ADCY3, adenylate cyclase 3; SH2B1, SH2B adaptor protein 1; TFAP2B, transcription factor AP-2 beta*.

### Association of *MSRA* rs545854 With Hyperuricemia

Since the frequencies of eating meat, hypertriglyceridemia, abnormal BMI, central obesity, hypertension, and NAFLD were unevenly distributed between the hyperuricemia and non-hyperuricemia groups, we included these variables as covariates in the model to exclude their potential confounding effects on the association of the SNP with hyperuricemia. According to the genetic frequencies of rs545854 in the hyperuricemia and non-hyperuricemia groups (Table 2), a dominant inheritance model was conducted. As shown in Table 3, the G allele of rs545854 was associated with an increased risk of hyperuricemia (*P* = 0.0188) after adjusting for eating meat, hypertension, high triglycerides, and NAFLD. Further adjustment for BMI category and central obesity did not diminish the association of the rs545854 G allele with hyperuricemia in Chinese men.

**Table 3:** The association of genotype with hyperuricemia in dominant model.

**Regression Models**	**Genotype**	**Hyperuricemia (*n =* 66)**	**Non-hyperuricemia (*n =* 408)**	**OR (95%CI)**^**a**^	***P*-value^**a**^**	**OR (95%CI)b**^**b**^	***P*-value**^**b**^
Dominant	CC	7 (10.61%)	90 (22.06%)	1.00 (reference)	**0.0188**	1.00 (reference)	**0.0196**
	CG+GG	59 (89.39%)	318 (77.94%)	2.80 (1.19–6.64)		2.81 (1.18–6.70)	

*Data are expressed as n (%) or 95% Confidence Interval. Bold values indicate statistical significance*.

a *Adjusted for eating meat, hypertension, hypertriglyceridemia, NAFLD*.

b *Adjusted for eating meat, hypertension, hypertriglyceridemia, NAFLD, BMI category, central obesity*.

### Stratified Analyses

**Figure 1:**
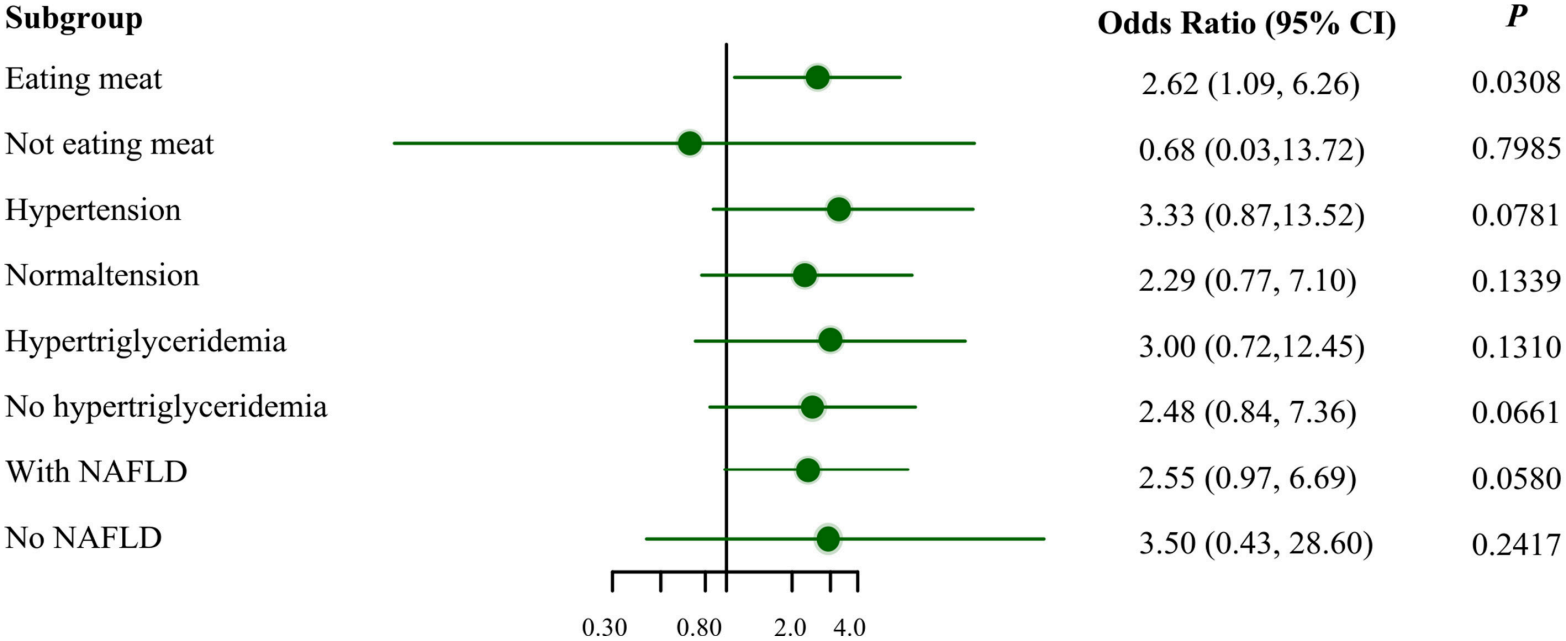
Subgroup analysis for the association of *MSRA* rs545854 with hyperuricemia. *NAFLD*, non-alcoholic fatty liver disease. All analyses were adjusted for eating meat, hypertension, hypertriglyceridemia, NAFLD, BMI category, central obesity, with the exception of subgroup variables.

Figure 1 illustrates the association between *MSRA* rs545854 with hyperuricemia according to subgroups of men based on meat eating, hypertension, hypertriglyceridemia, and NAFLD. The association between *MSRA* rs545854 and hyperuricemia remained significant in the meat-eating group [odds ratio (OR) = 2.62, 95% confidence interval (CI) = 1.09–6.26, *P* = 0.0308], whereas the association was not observed in the other subgroups.

### Power Analysis

The 1,000 Genomes Project data indicate that the minor frequency of rs545854 is 0.354 in the Han Chinese population of Beijing. In addition, a recent meta-analysis reported that the prevalence of hyperuricemia in Chinese men was 19.4% (8). Considering this information, the sample size of this study had 99.99% power to detect significant effects with the OR of 2.81 obtained in this study (two-tailed test with α = 0.05).

## Discussion

Among the 47 SNPs reported to be related to MS components, we identified that rs545854 in the *MSRA* gene independently affects the SUA level and increases the risk of hyperuricemia in Chinese men. This effect remained pronounced in a subgroup analysis among only meat eaters.

Although numerous longitudinal studies have indicated an association of hyperuricemia with multiple diseases, including obesity and MS, the potential genetic evidence of this relationship has thus far remained unclear. To our best knowledge, this is the first study to assess the correlation of obesity susceptibility loci with the level of SUA and the risk of hyperuricemia in Chinese men. Our results suggest that this association could be related to a shared genetic background, which can help further elucidate the underlying mechanism and related biological pathways. Since this design failed to fulfill one of the conditions of Mendelian randomization analysis, i.e., an obesity-related SNP should not influence the hyperuricemia risk via pathways other than those related to obesity (25, 26), we only analyzed the association of obesity genetic variations with hyperuricemia risk, rather than determining the causal relationship between them.

The SNP rs545854, originally named rs7826222, is located ~50,000 bp upstream of the *MSRA* gene (27, 28), which encodes a protein that protects against oxidative stress by repairing methionine (29). Rs545854 was previously reported to be associated with body fat distribution traits, indicating that carriers of the *MSRA* rs545854 risk allele (G-allele) tended to have a considerably lower leg fat percentage with a trend toward a higher trunk fat percentage, trunk-periphery fat ratio, and visceral fat area percentage adjusted for total fat (30). In addition, large-scale genome-wide association studies confirmed that the G-allele of *MSRA* rs545854 was associated with greater WC and BMI (31–33). Moreover, index variants near or at *MSRA* were found to contribute to obesity in the Han Chinese population (28). Thus, we further extend the risk of the G-allele of the *MSRA* rs545854 polymorphism for increasing SUA and contributing to hyperuricemia. Recent studies indicated associations of central obesity-associated variants in *MSRA* with metabolic traits, lower fasting levels of serum insulin, and decreased homeostatic model assessment-insulin resistance among men (34, 35). Hyperinsulinemia, a consequence of overweight and obesity, was shown to enhance the renal proximal tubular reabsorption of SUA, leading to elevation of SUA levels (36), which could explain the mechanisms underlying the associations between *MSRA* rs545854 and hyperuricemia. Consequently, the present results may be in line with a previous bidirectional Mendelian randomization study suggesting that elevated SUA is a consequence rather than a cause of adiposity in adult Caucasians (26).

There is further evidence that may shed some light on the association between obesity and hyperuricemia. First, obese individuals tend to have elevated levels of SUA due to higher urinary excretion and reduced SUA clearance in comparison with individuals of normal weight (37, 38). Second, weight loss in obese subjects was reported to be accompanied by a decrease in both SUA levels and xanthine oxidoreductase (XOR) activity (39, 40); XOR is responsible for the breakdown of hypoxanthine and xanthine into SUA. Third, animal experiments suggested that the underlying mechanisms of elevated SUA in obese adipose tissue may result from dysregulation of adipocytokines and chronic low-grade inflammation (41–43).

Results of the stratified analysis indicated a statistically significant association between *MSRA* rs545854 with hyperuricemia in meat eaters. Both genetic and dietary factors have been shown to influence the SUA level (44–48). Moreover, a large cohort study recently confirmed that low intake of red meat could reduce SUA levels during 26 years of follow-up (49). These findings suggest that males harboring the *MSRA* rs545854 SNP should limit their meat intake to prevent hyperuricemia.

The main strength of this study is that it is the first assessment of the correlation of obesity susceptibility loci with both SUA level and risk of hyperuricemia in Chinese men, indicating a significant association for *MSRA* rs545854. Thus, these findings shed light on the underlying biological mechanisms mediating obesity and hyperuricemia. However, there are some limitations of our study. First, there could be recall bias while filling out the questionnaire in this study. Second, we only focused on the association between MS-or obesity-associated SNPs and hyperuricemia in Chinese men, since metabolic or additional hormonal effects could impact the SUA levels and hyperuricemia in women. But further studies should investigate the association in women. Third, we did not use Mendelian randomization to validate this association between SNPs and hyperuricemia, and a significant association could be affected by other potential confounding factors. Indeed, a significant association was only observed in the meat-eating group, whereas no association was found in a series of stratified analyses based on hypertension, hypertriglyceridemia, and NAFLD. Hence, these stratified analyses indicated that there are potentially other confounding factors influencing the association, including hypertension, hypertriglyceridemia, and NAFLD as well as some other groups of foods that may be more likely to cause hyperuricemia (e.g., seafood). Hence, further study needs to consider the effect of some other groups of food on hyperuricemia. Fourth, this association should be confirmed in other ethnic groups to understand the genetic diversity and common features contributing to these associations. Gaining a greater understanding of these mechanisms can help guide improved prevention strategies to reduce the incidence of hyperuricemia tailored to specific populations and genetic backgrounds.

## Conclusion

This study suggested that an obesity-related genetic variant (*MSRA* rs545854) was associated with SUA level and the risk of hyperuricemia in Chinese men. *MSRA* rs545854 was associated with the risk of hyperuricemia independent of BMI and central obesity. Additionally, our results suggest that male carriers of *MSRA* rs545854 might be able to limit their meat intake to prevent hyperuricemia.

## Ethics Statement

The study protocol was approved by the ethics committee of Fu Xing Hospital (Beijing, China), and written informed consent was provided by each participant.

## Author Contributions

ZM performed the stratified analysis, interpreted the results, and drafted the manuscript. YW contributed to the study idea and data analysis. CX and JP assisted in interpreting the findings and revising the manuscript. LH and JW recruited study participants and performed the clinical diagnoses. FA, YZ, MY, and SZ were responsible for data collection and the Hardy-Weinberg equilibrium test. XY contributed to the study concept and design as well as revision of the manuscript. All authors read and approved the final manuscript.

### Conflict of Interest Statement

The authors declare that the research was conducted in the absence of any commercial or financial relationships that could be construed as a potential conflict of interest.

## Abbreviations

SUA: serum uric acid
SNPs: single nucleotide polymorphisms
BMI: body mass index
MS: metabolic syndrome
WC: waist circumference
TC: total cholesterol
LDL-C: low-density lipoprotein cholesterol
HDL-C: high-density lipoprotein cholesterol
SNP: single-nucleotide polymorphisms
MALDI-TOF-MS: matrix-assisted laser desorption ionization time-of-flight mass spectrometry
*LY86*: lymphocyte antigen 86
*MC4R*: melanocortin 4 receptor
*MSRA*: methionine sulfoxide reductase
*ADCY3*: adenylate cyclase 3
*SH2B1*: SH2B adaptor protein 1
*TFAP2B*: transcription factor AP-2 beta
XOR: xanthine oxido reductase
NAFLD: nonalcoholic fatty liver disease.
