# Supplementary material for: Obesity-Related Genetic Variants and Hyperuricemia Risk in Chinese Men
Source: Front Endocrinol (Lausanne). 2019 Apr 12;10:230. doi: 10.3389/fendo.2019.00230 (PMC6474097; doi:10.3389/fendo.2019.00230)
Supplement: Supplementary file 1 [file Table_1.docx]

### Table S1| Association of 41 SNPs with SUA

| **SNP** | **Locus** | **Genotype** | **Case** | **Control** | ***X^2^*** | ***P*** | **SUA level** | ***F*** | ***P*** | ***P_het_*** |
| --- | --- | --- | --- | --- | --- | --- | --- | --- | --- | --- |
| rs1011731 | *DNM3* | TT | 49 (13.8) | 306 (86.2) | 0.054 | 0.9736 | 333.35 ± 75.23 | 0.10 | 0.9046 | 0.92 |
|  |  | TC | 16 (14.68) | 93 (85.32) |  |  | 334.57 ± 69.85 |  |  |  |
|  |  | CC | 1 (14.29) | 6 (85.71) |  |  | 321.71 ± 87.06 |  |  |  |
| rs10195252 | *GRB14* | TT | 52 (14.29) | 312 (85.71) | 1.853 | 0.3960 | 332.20 ± 74.40 | 0.31 | 0.7325 | 0.29 |
|  |  | TC | 14 (14.74) | 81 (85.26) |  |  | 338.57 ± 75.33 |  |  |  |
|  |  | CC | 0 (0.00) | 11 (100.00) |  |  | 327.64 ± 54.01 |  |  |  |
| rs10767664 | *BDNF* | AA | 20 (15.5) | 109 (84.5) | 0.27 | 0.7562 | 334.85 ± 78.19 | 0.27 | 0.7672 | 0.41 |
|  |  | AT | 35 (14.11) | 213 (85.89) |  |  | 331.12 ± 74.06 |  |  |  |
|  |  | TT | 11 (11.83) | 82 (88.17) |  |  | 337.23 ± 68.71 |  |  |  |
| rs10968576 | *LRRN6C* | AA | 49 (13.88) | 304 (86.12) | NA | 0.9097* | 335.59 ± 74.74 | 0.60 | 0.5508 | **0.06** |
|  |  | AG | 17 (14.66) | 99 (85.34) |  |  | 326.92 ± 72.35 |  |  |  |
|  |  | GG | 0 (0.00) | 2 (100.00) |  |  | 335.00 ± 46.67 |  |  |  |
| rs11084753 | *KCTD15* | AA | 30 (15.23) | 167 (84.77) | 0.810 | 0.6673 | 337.84 ± 78.02 | 0.59 | 0.5556 | 0.99 |
|  |  | AG | 30 (14.02) | 184 (85.98) |  |  | 330.03 ± 73.29 |  |  |  |
|  |  | GG | 6 (10.53) | 51 (89.47) |  |  | 335.74 ± 61.82 |  |  |  |
| rs11191580 | *NT5C2* | TT | 34 (13.88) | 211 (86.12) | 0.264 | 0.8763 | 336.47 ± 69.69 | 2.47 | 0.0860 | 0.97 |
|  |  | CT | 28 (14.66) | 163 (85.34) |  |  | 334.08 ± 75.89 |  |  |  |
|  |  | CC | 4 (11.43) | 31 (88.57) |  |  | 306.97 ± 88.62 |  |  |  |
| rs12229654 | *MYL2* | TT | 55 (15.28) | 305 (84.72) | 3.366 | 0.1858 | 338.12 ± 73.08 | 3.52 | 0.0304 | 0.88 |
|  |  | GT | 9 (8.74) | 94 (91.26) |  |  | 316.44 ± 75.63 |  |  |  |
|  |  | GG | 2 (22.22) | 7 (77.78) |  |  | 340.56 ± 68.96 |  |  |  |
| rs1424233 | *MAF* | AA | 32 (15.53) | 174 (84.47) | 0.746 | 0.6886 | 337.76 ± 79.53 | 0.73 | 0.4818 | 0.94 |
|  |  | GA | 27 (12.92) | 182 (87.08) |  |  | 328.99 ± 69.37 |  |  |  |
|  |  | GG | 7 (12.28) | 50 (87.72) |  |  | 334.13 ± 69.63 |  |  |  |

| SNP | Locus | Genotype | Case | Control | *X^2^* | *P* | SUA level | *F* | *P* | *P_het_* |
| --- | --- | --- | --- | --- | --- | --- | --- | --- | --- | --- |
| rs1558902 | *FTO* | TT | 52 (13.87) | 323 (86.13) | 0.015 | 0.9927 | 332.43 ± 72.68 | 0.19 | 0.8266 | **<0.0001** |
|  |  | TA | 12 (14.29) | 72 (85.71) |  |  | 337.79 ± 77.91 |  |  |  |
|  |  | AA | 2 (13.33) | 13 (86.67) |  |  | 330.40 ± 90.80 |  |  |  |
| rs16933812 | *PAX5* | TT | 31 (12.02) | 227 (87.98) | 2.083 | 0.3530 | 329.06 ± 76.68 | 1.13 | >0.05 | 0.88 |
|  |  | GT | 31 (16.85) | 153 (83.15) |  |  | 338.49 ± 69.28 |  |  |  |
|  |  | GG | 4 (13.79) | 25 (86.21) |  |  | 343.14 ± 77.53 |  |  |  |
| rs17782313 | *MC4R* | TT | 23 (14.29) | 138 (85.71) | 1.445 | 0.4855 | 335.42 ± 72.73 | 1.75 | >0.05 | **<0.0001** |
|  |  | TC | 39 (13.22) | 256 (86.78) |  |  | 330.79 ± 74.02 |  |  |  |
|  |  | CC | 4 (23.53) | 13 (76.47) |  |  | 364.53 ± 81.17 |  |  |  |
| rs2074356 | *HECTD4* | CC | 55 (14.95) | 313 (85.05) | 3.310 | 0.1914 | 337.78 ± 75.08 | 3.22 | 0.0410 | 0.98 |
|  |  | TC | 9 (9.28) | 88 (90.72) |  |  | 316.59 ± 66.61 |  |  |  |
|  |  | TT | 2 (28.57) | 5 (71.43) |  |  | 341.14 ± 90.88 |  |  |  |
| rs2112347 | *POC5* | GG | 20 (13.89) | 124 (86.11) | 1.947 | 0.3777 | 341.85 ± 75.90 | 1.74 | 0.1759 | 0.63 |
|  |  | GT | 38 (15.97) | 200 (84.03) |  |  | 333.36 ± 74.34 |  |  |  |
|  |  | TT | 8 (9.76) | 74 (90.24) |  |  | 322.89 ± 69.34 |  |  |  |
| rs2228570 | *FokI* | TT | 32 (13.45) | 206 (86.55) | 4.527 | 0.1040 | 333.58 ± 72.50 | 0.13 | 0.8790 | <**0.0001** |
|  |  | TC | 8 (8.89) | 82 (91.11) |  |  | 332.52 ± 67.44 |  |  |  |
|  |  | CC | 26 (18.71) | 113 (81.29) |  |  | 337.00 ± 80.82 |  |  |  |
| rs2237892 | *KCNQ1* | CC | 34 (15.25) | 189 (84.75) | 0.906 | 0.6359 | 336.16 ± 76.48 | 0.92 | 0.3973 | 0.32 |
|  |  | TC | 26 (13.47) | 167 (86.53) |  |  | 333.36 ± 72.10 |  |  |  |
|  |  | TT | 6 (10.53) | 51 (89.47) |  |  | 321.19 ± 71.72 |  |  |  |
| rs2241423 | *MAP2K5* | AA | 21 (12.80) | 143 (87.20) | 0.845 | 0.6555 | 349.58 ±70.70 | 0.69 | 0.1184 | 0.59 |
|  |  | GA | 33 (14.04) | 202 (85.96) |  |  | 333.48 ±73.26 |  |  |  |
|  |  | GG | 12 (17.39) | 57 (82.61) |  |  | 327.66 ±75.90 |  |  |  |
| rs2535633 | *ITIH4* | GG | 24 (17.52) | 113 (82.48) | 2.537 | 0.2813 | 336.04 ± 77.72 | 0.44 | 0.6431 | 0.48 |
|  |  | GC | 32 (13.56) | 204 (86.44) |  |  | 334.57 ± 73.61 |  |  |  |
|  |  | CC | 10 (10.31) | 87 (89.69) |  |  | 327.33 ± 69.81 |  |  |  |
| SNP | Locus | Genotype | Case | Control | *X^2^* | *P* | SUA level | *F* | *P* | *P_het_* |
| rs2605100 | *LYPLAL1* | GG | 39 (14.13) | 237 (85.87) | 0.456 | 0.7963 | 330.98 ± 74.19 | 1.22 | 0.2969 | 0.97 |
|  |  | AG | 24 (14.63) | 140 (85.37) |  |  | 339.74 ± 74.31 |  |  |  |
|  |  | AA | 3 (10.00) | 27 (90.00) |  |  | 320.30 ± 71.26 |  |  |  |
| rs2815752 | *NEGR1* | TT | 58 (14.5) | 342 (85.5) | 1.18 | 0.6908 | 335.55 ± 73.58 | 1.18 | 0.3097 | 0.87 |
|  |  | CT | 8 (11.59) | 61 (88.41) |  |  | 323.75 ± 76.42 |  |  |  |
|  |  | CC | 0 (0.00) | 2 (100.00) |  |  | 285.50 ± 12.02 |  |  |  |
| rs2867125 | *TMEM18* | GG | 54 (14.29) | 324 (85.71) | NA | 0.6626* | 335.80 ± 73.47 | 0.97 | 0.3783 | 0.99 |
|  |  | GA | 11 (12.36) | 78 (87.64) |  |  | 323.90 ± 75.47 |  |  |  |
|  |  | AA | 1 (20.00) | 4 (80.00) |  |  | 324.00 ± 86.38 |  |  |  |
| rs2943650 | *IRS1* | TT | 55 (13.82) | 343 (86.18) | 0.108 | 0.7430 | 334.81 ± 73.82 | 1.01 | 0.3154 | 0.20 |
|  |  | TC | 11 (15.28) | 61 (84.72) |  |  | 325.28 ± 75.58 |  |  |  |
| rs3810291 | *ZC3H4* | GG | 38 (15.51) | 207 (84.49) | 1.146 | 0.5638 | 338.69 ± 74.72 | 1.95 | 0.1429 | 0.01 |
|  |  | AG | 22 (13.10) | 146 (86.90) |  |  | 330.82 ± 74.18 |  |  |  |
|  |  | AA | 6 (10.53) | 51 (89.47) |  |  | 318.07 ± 69.79 |  |  |  |
| rs3817334 | *MTCH2* | CC | 33 (14.29) | 198 (85.71) | 0.034 | 0.9832 | 332.19 ± 74.79 | 0.70 | 0.4973 | 0.57 |
|  |  | TC | 26 (13.68) | 164 (86.32) |  |  | 337.24 ± 69.30 |  |  |  |
|  |  | TT | 7 (14.29) | 42 (85.71) |  |  | 323.76 ± 88.11 |  |  |  |
| rs4823006 | *ZNRF3* | GG | 20 (14.71) | 116 (85.29) | 1.345 | 0.5104 | 335.35 ± 79.02 | 0.35 | 0.7071 | 0.96 |
|  |  | GA | 35 (15.02) | 198 (84.98) |  |  | 334.51 ± 75.24 |  |  |  |
|  |  | AA | 11 (10.48) | 94 (89.52) |  |  | 328.04 ± 64.83 |  |  |  |
| rs4846567 | *LYPLAL1* | GG | 32 (14.41) | 190 (85.59) | 0.130 | 0.9370 | 328.51 ± 78.42 | 1.25 | 0.2876 | 0.99 |
|  |  | GT | 27 (13.37) | 175 (86.63) |  |  | 339.65 ± 67.87 |  |  |  |
|  |  | TT | 7 (14.89) | 40 (85.11) |  |  | 330.21 ± 77.76 |  |  |  |
| rs534870 | *SPRY2* | GG | 27 (15.52) | 147 (84.48) | 2.416 | 0.2987 | 335.23 ± 76.76 | 0.26 | 0.7737 | 0.55 |
|  |  | GA | 34 (14.47) | 201 (85.53) |  |  | 333.83 ± 74.03 |  |  |  |
|  |  | AA | 5 (7.81) | 59 (92.19) |  |  | 327.53 ± 67.34 |  |  |  |

| SNP | Locus | Genotype | Case | Control | *X^2^* | *P* | SUA level | *F* | *P* | *P_het_* |
| --- | --- | --- | --- | --- | --- | --- | --- | --- | --- | --- |
| rs541874 | *LOC107987166* | TT | 18 (13.95) | 111 (86.05) | 0.144 | 0.9307 | 334.57 ± 73.08 | 0.04 | 0.9579 | 0.99 |
|  |  | CT | 32 (13.56) | 204 (86.44) |  |  | 332.46 ± 75.44 |  |  |  |
|  |  | CC | 16 (15.09) | 90 (84.91) |  |  | 334.33 ± 72.72 |  |  |  |
| rs571312 | *NT5C2* | GG | 37 (12.21) | 266 (87.79) | 4.295 | 0.1168 | 329.90 ± 72.98 | 2.55 | 0.0792 | 0.36 |
|  |  | GT | 25 (15.82) | 133 (84.18) |  |  | 336.42 ± 73.38 |  |  |  |
|  |  | TT | 4 (30.77) | 9 (69.23) |  |  | 375.15 ± 98.09 |  |  |  |
| rs671 | *ALDH2* | GG | 52 (15.12) | 292 (84.88) | 1.880 | 0.3907 | 338.70 ± 73.72 | 3.29 | 0.0382 | 0.96 |
|  |  | GA | 12 (10.26) | 105 (89.74) |  |  | 318.70 ± 72.05 |  |  |  |
|  |  | AA | 2 (18.18) | 9 (81.82) |  |  | 325.45 ± 86.67 |  |  |  |
| rs6784615 | *NISCH* | TT | 63 (14.13) | 383 (85.87) | 0.000 | 0.7658 | 333.36 ± 74.36 | 0.02 | 0.8996 | 0.84 |
|  |  | CT | 3 (12.00) | 22 (88.00) |  |  | 335.28 ± 69.54 |  |  |  |
| rs6795735 | *ADAMTS9* | TT | 23 (12.99) | 154 (87.01) | 1.682 | 0.4312 | 330.12 ± 70.18 | 2.48 | 0.0847 | <0.0001 |
|  |  | CT | 41 (15.41) | 225 (84.59) |  |  | 338.39 ± 75.65 |  |  |  |
|  |  | CC | 2 (7.14) | 26 (92.86) |  |  | 307.68 ± 78.50 |  |  |  |
| rs6861681 | *CPEB4* | GG | 54 (13.92) | 334 (86.08) | NA | 0.8170* | 334.09 ± 74.44 | 0.27 | 0.7669 | 0.99 |
|  |  | GA | 11 (13.92) | 68 (86.08) |  |  | 332.84 ± 71.69 |  |  |  |
|  |  | AA | 1 (25.00) | 3 (75.00) |  |  | 307.25 ± 89.27 |  |  |  |
| rs6905288 | *VEGFA1* | AA | 33 (13.25) | 216 (86.75) | NA | 0.7311* | 332.59 ±71.20 | 1.12 | 0.3263 | 0.81 |
|  |  | AG | 26 (14.21) | 157 (85.79) |  |  | 337.25 ±76.26 |  |  |  |
|  |  | GG | 7 (17.95) | 32 (82.05) |  |  | 317.80 ±83.48 |  |  |  |
| rs7138803 | *FAIM2* | GG | 27 (11.84) | 201 (88.16) | 2.017 | 0.3647 | 330.79 ± 68.89 | 0.71 | 0.4925 | 0.55 |
|  |  | AG | 32 (16.67) | 160 (83.33) |  |  | 334.10 ± 80.05 |  |  |  |
|  |  | AA | 7 (13.73) | 44 (86.27) |  |  | 344.37 ± 72.26 |  |  |  |
| rs7566605 | *INSIG2* | GG | 31 (17.13) | 150 (82.87) | 2.524 | 0.2830 | 342.09 ± 79.90 | 1.89 | 0.1519 | 0.01 |
|  |  | GC | 29 (11.74) | 218 (88.26) |  |  | 328.20 ± 69.29 |  |  |  |
|  |  | CC | 6 (13.64) | 38 (86.36) |  |  | 330.68 ± 72.18 |  |  |  |

| SNP | Locus | Genotype | Case | Control | *X^2^* | *P* | SUA level | *F* | *P* | *P_het_* |
| --- | --- | --- | --- | --- | --- | --- | --- | --- | --- | --- |
| rs8050136 | *FTO* | AA | 12 (14.12) | 73 (85.88) | 0.005 | 0.9974 | 333.79 ± 93.24 | 0.14 | 0.8696 | <0.0001 |
|  |  | CA | 52 (13.87) | 323 (86.13) |  |  | 337.14 ± 77.68 |  |  |  |
|  |  | CC | 2 (14.29) | 12 (85.71) |  |  | 332.43 ± 72.68 |  |  |  |
| rs9356744 | *CDKAL1* | TT | 21 (12.28) | 150 (87.72) | 1.371 | 0.5039 | 325.82 ± 75.33 | 1.92 | 0.1472 | 0.99 |
|  |  | CT | 36 (16.00) | 189 (84.00) |  |  | 340.14 ± 73.40 |  |  |  |
|  |  | CC | 9 (12.16) | 65 (87.84) |  |  | 329.74 ± 72.01 |  |  |  |
| rs9816226 | *ETV5* | TT | 61 (13.71) | 384 (86.29) | NA | 0.6019* | 332.01 ± 74.66 | 1.63 | 0.1976 | <0.0001 |
|  |  | TA | 5 (20.00) | 20 (80.00) |  |  | 358.88 ± 59.93 |  |  |  |
|  |  | AA | 0 (0.00) | 3 (100.00) |  |  | 317.33 ± 95.71 |  |  |  |
| rs984222 | *TBX15* | GG | 23 (12.04) | 168 (87.96) | 1.720 | 0.4232 | 329.68 ± 73.99 | 3.98 | 0.0193 | 0.92 |
|  |  | CG | 31 (14.49) | 183 (85.51) |  |  | 329.65 ± 71.62 |  |  |  |
|  |  | CC | 12 (18.46) | 53 (81.54) |  |  | 357.45 ± 79.20 |  |  |  |
| rs9939609 | *FTO* | TT | 52 (14.02) | 319 (85.98) | 0.031 | 0.9846 | 332.30 ± 72.98 | 0.16 | 0.8518 | <0.0001 |
|  |  | TA | 12 (13.79) | 75 (86.21) |  |  | 336.92 ± 76.79 |  |  |  |
|  |  | AA | 2 (12.50) | 14 (87.50) |  |  | 337.31 ± 88.55 |  |  |  |

### Data are expressed as mean ± SD or n (%); NA, no analysis; * Fisher's exact test.

### *P_het,_ P* for heterogeneity; *DNM3*, dynamin 3; *GRB14*, growth factor receptor bound protein 14; [*BDNF*, brain derived neurotrophic factor](https://www.ncbi.nlm.nih.gov/gene/627); [*LRRN6C*, leucine rich repeat and Ig domain containing 2](https://www.ncbi.nlm.nih.gov/gene/158038); [*KCTD15*, potassium channel tetramerization domain containing 15](https://www.ncbi.nlm.nih.gov/gene/79047); *NT5C2*, nucleotidase, cytosolic II; *MYL2*, Myosin Regulatory Light Chain; MAF, minor allele frequency; TNNI3K, cardiac troponin I-interacting kinase; *FTO*, fat mass and obesity associated; *PAX5*, paired-box 5; *MC4R*, melanocortin 4 receptor; [*HECTD4*, HECT domain E3 ubiquitin protein ligase 4](https://www.ncbi.nlm.nih.gov/gene/283450); *POC5*, protein of centriole 5; [*KCNQ1*, KQT‑like subfamily Q, member 1](https://www.ncbi.nlm.nih.gov/gene/3784); *MAP2K5*, mitogen-activated protein kinase kinase 5; [*ITIH4*, inter-alpha-trypsin inhibitor heavy chain family member 4](https://www.ncbi.nlm.nih.gov/gene/3700); *LYPLAL1*, lysophospholipase like 1; *NEGR1*, neuronal growth regulator 1; *IRS1*, insulin rceptor substrate-1; *MTCH2,*mitochondrial carrier homolog 2; *ZC3H4*, [zinc finger CCCH-type containing 4](https://www.ncbi.nlm.nih.gov/gene/23211); [*ZNRF3*, zinc and ring finger 3](https://www.ncbi.nlm.nih.gov/gene/84133); [*LYPLAL1*, lysophospholipase like 1](https://www.ncbi.nlm.nih.gov/gene/127018); [*SPRY2*, sprouty RTK signaling antagonist 2](https://www.ncbi.nlm.nih.gov/gene/10253); *MSRA*, methionine sulfoxide reductase A; [*ALDH2*, aldehyde dehydrogenase 2 family member](https://www.ncbi.nlm.nih.gov/gene/217); *NISCH*, neonatal ichthyosis sclerosing cholangitis; *ADAMTS9*, A disintegrin-like and metalloprotease with thrombospondin type I motifs; *CPEB4*, cytoplasmic polyadenylation element binding protein 4; *VEFGFA1*, vascular endothelial growth factor A; [*FAIM2*, Fas apoptotic inhibitory molecule 2](https://www.ncbi.nlm.nih.gov/gene/23017); *INSIG2*, insulin-induced gene 2; *CDKAL1*, cyclin-dependent kinase 5 regulatory; *ETV5*, ETS variant 5; [*TBX15*,T-box 15](https://www.ncbi.nlm.nih.gov/gene/6913).
